# Effect of Sunlight Radiation on the Growth and Chemical Constituents of *Salvia plebeia* R.Br.

**DOI:** 10.3390/molecules22081279

**Published:** 2017-08-01

**Authors:** Hyun-Jae Jang, Seung-Jae Lee, Cha Young Kim, Joo Tae Hwang, Jung Ho Choi, Jee Hun Park, Seung Woong Lee, Mun-Chual Rho

**Affiliations:** 1 Immunoregulatory Material Research Center, Korea Research Institute of Bioscience and Biotechnology, 181 Ipsin-gil, Jeongeup-si, Jeonbuk 56212, Korea; water815@kribb.re.kr (H.-J.J.); seung99@kribb.re.kr (S.-J.L.); jthwang@kiom.re.kr (J.T.H.); 2 Biological Resource Center, Korea Research Institute of Bioscience and Biotechnology, 181 Ipsin-gil, Jeongeup-si, Jeonbuk 56212, Korea; kimcy@kribb.re.kr; 3 R&D Center, Korean Drug Co., Ltd., Seoul 06300, Korea; bearlss@hanmail.net (J.H.C.); wlgns1010@hanmail.net (J.H.P.)

**Keywords:** *Salvia plebeia*, HPLC validation, triterpenoid, flavonoid, antioxidant, cultivation, UV

## Abstract

This study investigated the chemical composition changes of *Salvia plebeia* R.Br. cultivated under different light sources, including florescent light and sunlight. The plants were exposed to fluorescent light for four months and sunlight and then examined for the next 5–7 months. Plants were harvested monthly during the seven months, and we examined whether the difference in light source affected the phenolic and flavonoid contents and antioxidant activity. A simple and reliable HPLC method using a PAH C_18_ column was applied for the quantitative analysis of two triterpenoids from the *S. plebeia* groups. Oleanolic acid (OA) and ursolic acid (UA) showed good linearity (*R*^2^ > 0.9999) within the test ranges (0.005–0.05 mg/mL), and the average percentage recoveries of the OA and UA were 95.1–104.8% and 97.2–107.1%, respectively. The intra- and inter-day relative standard deviations (RSDs) were less than 2.0%. After exposure to sunlight, the phenolic contents, including rosmarinic acid, showed a reduced tendency, whereas the flavonoid contents, including homoplantaginin and luteolin 7-glucoside, were increased. The content of the triterpenoids also showed an increased tendency under sunlight irradiation, but the variance was not larger than those of the phenolic and flavonoid contents. Among experimental groups, the group harvested at six months, having been exposed to sunlight for two months, showed the most potent antioxidant activity. Therefore, these results showed that the chemical composition and antioxidant activities of *S. plebeia* R.Br. was affected from environmental culture conditions, such as light source. Our studies will be useful for the development of functional materials using *S. plebeia* R.Br.

## 1. Introduction

Famers in various regions of Korea cultivate numerous dietary health foods or medicinal crops. Above all, *Salvia plebeia* R.Br. has received attention from many consumers because it is a promising therapeutic agent for various diseases and conditions, such as inflammation, arthritis, atopic dermatitis, asthma, hepatitis, and gastric ulcers [1,2,3,4,5]. *S. plebeia* is a member of the family Lamiaceae and is an annual or biennial plant that is widely distributed throughout the world, including in Korea and China [6]. *S. plebeia* contains diverse phytochemical constituents, such as terpenoids [7,8], flavonoids [9] and phenolic compounds [10], that contribute to its pharmacological activities, including antiviral, antimicrobial, anti-cancer, antioxidant, and anti-inflammatory activities [6,7,8,9].

The consistent biological activity of a medicinal herb is related to the concentration ratio of the bioactive chemical components of the herb [11]. However, many environmental or other factors, such as the origin of the herb, harvesting time, climate, preservation method, and cultivation conditions, alter the chemical composition of the herbs [12]. Therefore, the therapeutic efficacy and safety of medicinal plants used in a specific patient population should be verified by an appropriate quality control method [11]. The HPLC method was previously established for chemical fingerprinting and quantitative analysis of *S. plebeia* [13], and the literature shows the chemical composition of *S. plebeia* varies under different cultivation conditions [14]. However, the chemical composition variance with regard to the different environmental conditions of *S. plebeia* remain unexplored.

The aim of this study is to contribute to the understanding of the cultivation conditions for achieving the suitable chemical components of *S. plebeia*, and two of its metabolites, oleanolic acid (OA) and ursolic acid (UA), were used to verify the quality of this herb.

## 2. Results and Discussion

### 2.1. The Appearance of S. plebeia Cultivated under Different Growth Conditions

**Figure 1:**
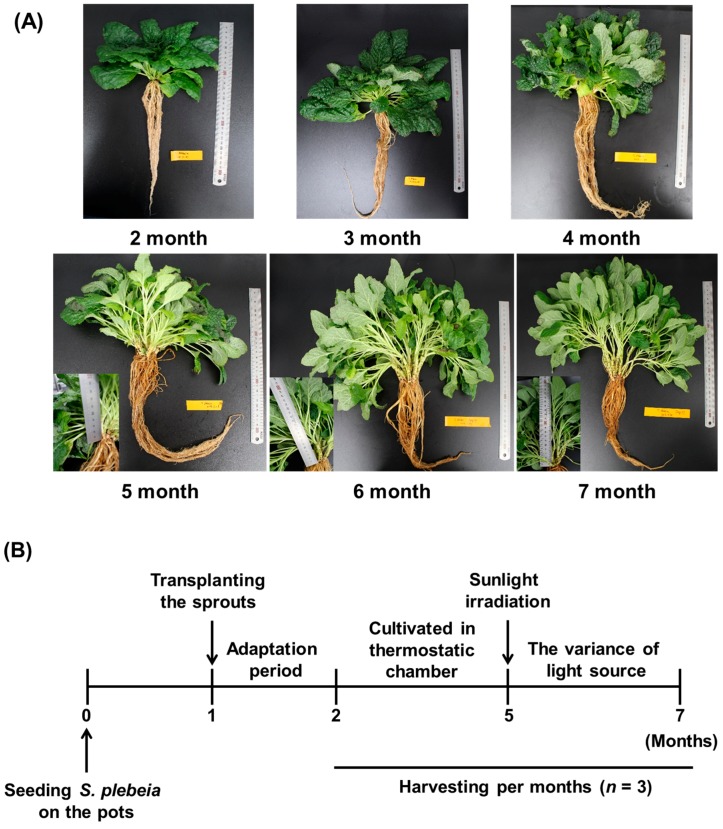
Appearance of *Salvia plebeia* cultivated under different light sources, including fluorescent light and natural sunlight (**A**) and the experimental design (**B**).

To investigate the effect of the environmental conditions on *S. plebeia*, we cultivated the plants in a thermostatic chamber, and then subjected the plants to different durations of fluorescent light and sunlight. The appearance of *S. plebeia* cultivated under the different conditions is shown in Figure 1. Compared to the groups (2, 3, and 4 months) grown under a fluorescent light source, the groups (5, 6, and 7 months) exposed to sunlight prior to harvesting had a flower stalk and bright green leaves and grew longer stem extensions. The average values of the flower stalk length were as follows: 2–3 months, not detectable; 4 months, <10 mm; 5 months, 48.5 ± 3.4 mm; 6 months, 75.3 ± 7.7 mm; and 7 months, 106.3 ± 5.2 mm (in the control plants). The groups (2, 3, and 4 months) cultured under fluorescent lights, which excluded the UV wavelength, had a lower biomass (weight of aerial parts) than the groups (5 and 6 months) cultured under sunlight irradiation. However, the last harvesting group (7 months), which was exposed to the longest duration of sunlight, showed a reduced biomass (2 months, 1.4 ± 0.5 g; 3 months, 4.8 ± 0.8 g; 4 months, 11.3 ± 1.8 g; 5 months, 13.1 ± 2.3 g; 6 months, 16.8 ± 2.0 g; and 7 months, 10.5 ± 0.9 g). In numerous studies, the ultraviolet radiation (UV) in sunlight results in mutagenic effects and physiological stresses in plants and therefore affects the contents of metabolites, biomass accumulation, and the growth of the plants [15,16]. According to previous literature, plants exposed to high-intensity light stress can be affected in photosynthetic CO_2_ fixation, and biomass accumulation decreases in full sunlight compared to moderate shade [17,18]. These results indicated that the biomass and growth of *S. plebeia* might be affected by the sunlight exposure duration.

### 2.2. Composition of Flavonoids and Phenolic Compounds in S. plebeia Depends on the Natural Sunlight Exposure Duration

**Figure 2:**
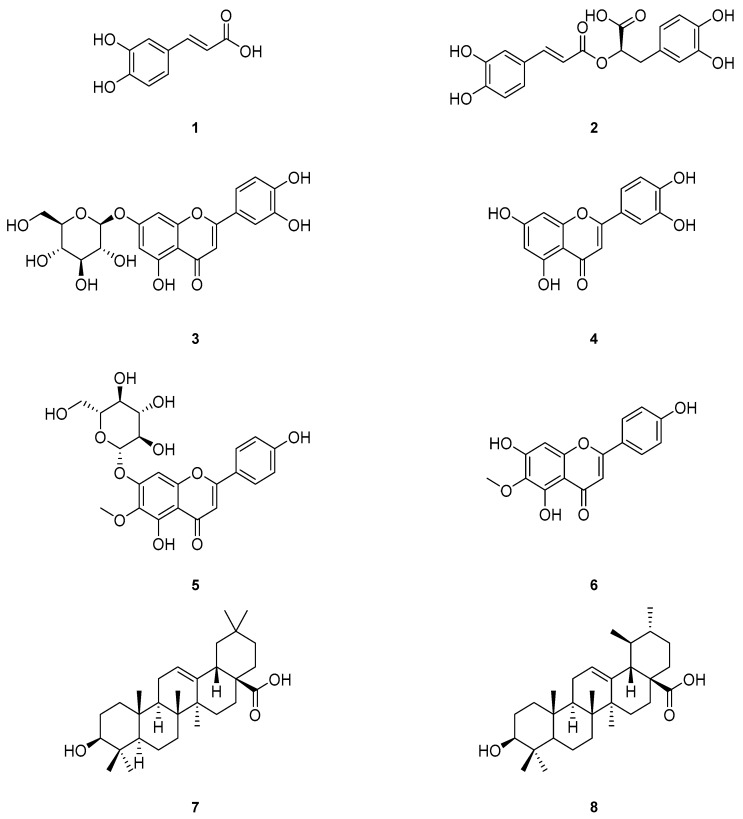
Chemical structures of the isolated compounds (**1**–**8**).

We found that *S. plebeia* obtained from various regions have different appearances, such as leaf shape and the elongation length of the flower stalk. Furthermore, *S. plebeia* samples obtained from various provinces in Korea showed different HPLC chromatogram patterns. The previously established HPLC analytical method and the optimal extraction condition [13] were slightly modified and applied in our studies. The contents of six chemical compounds (Figure 2), including caffeic acid (**1**), rosmarinic acid (**2**), luteolin 7-glucoside (**3**), luteolin (**4**), homoplantaginin (**5**), and hispidulin (**6**), were evaluated according to the HPLC analysis method, and the content variations of the chemical constituents were 0.58–1.19 (caffeic acid), 2.80–21.82 (luteolin 7-glucoside), 21.81–138.99 (rosmarinic acid), 6.57–53.13 (homoplantaginin), 0.62–1.57 (luteolin), and 0.80–2.79 mg/g (hispidulin) (Table 1). The content of the major compounds, luteolin 7-glucoside, rosmarinic acid, and homoplantaginin, varied considerably with differences of up to approximately eight-fold.

**Figure 3:**
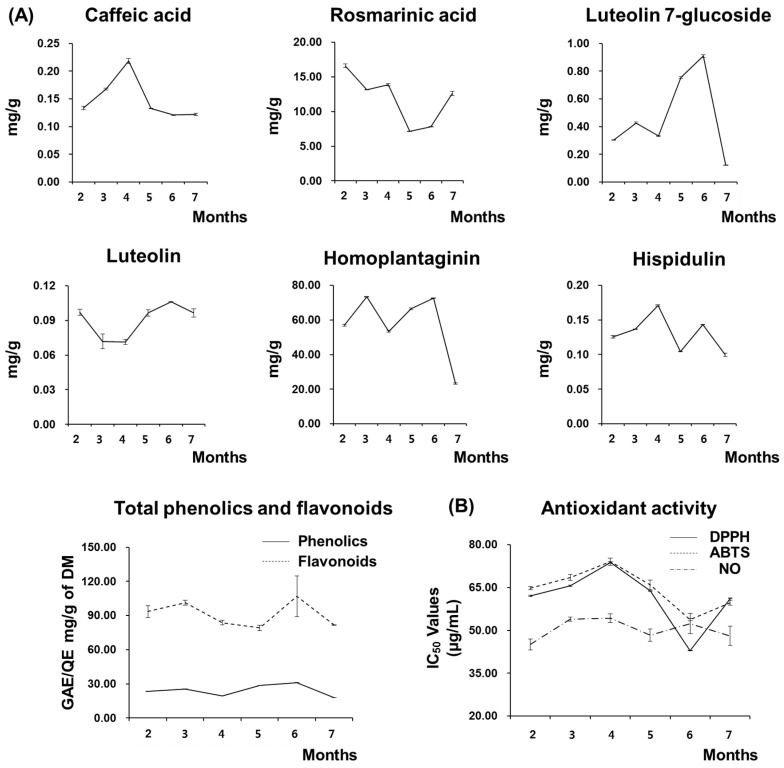
Total polyphenol and flavonoid contents (**A**) and radical scavenging activity (**B**) of *S. plebeia* in the different growing periods. The *S. plebeia* groups were sampled for a seven-month period (2, 3, and 4 months with fluorescent light; 5, 6, and 7 months with sunlight). The values represent the mean ± S.E. from triplicate experiments. (*DM:* Dry matter; *GAE:* Gallic acid equivalents; *QE:* Quercetin equivalents; *IC_50_:* concentration of 50% radical scavenging or anti-inflammatory activity).

The major constituents representing the phenolic and flavonoid compounds in *S. plebeia* are rosmarinic acid and homoplantaginin. As shown in Figure 3, a reduction in rosmarinic acid after altering the light source with sunlight was observed in the five- and six-month groups, whereas the homoplantaginin content was enhanced in the four- to six-month groups. Rosmarinic acid in the final harvesting group (seven months) had a similar content as in the three- and four-month groups grown under a fluorescent light source. However, the level of homoplantaginin was greatly reduced. Therefore, the acquisition of some metabolites from *S. plebeia* were related to the duration of exposure to sunlight. When compared with the total phenolic and total flavonoid contents (Figure 3), no remarkable variance of the total phenolic contents was shown among the other groups, and rosmarinic acid might be converted into other phenolic metabolites. However, the total flavonoid content showed a similar trend with regard to the content of the major flavonoid compounds homoplantaginin and luteolin 7-glucoside. According to Jin et al. [13], *S. plebeia* voucher specimens from different material sources indicate chemical variations of the plant constituents. Similar to previous studies, the chemical constituents of *S. plebeia* can be changed depending on the cultivation environment.

Rosmarinic acid is a well-known phytochemical constituent in the Lamiaceae family [19] and shows a variety of pharmacological properties, including anti-allergy and antioxidant activities [20,21]. The content of this phenolic compound is affected by the light intensity [22], harvesting time [23], light quality conditions [24], and drying methods [25], such as drying in the sunshine or shade or drying in a thermal condition (60 and 80 °C). In addition, many studies have indicated that UV-B irradiation in sunlight is involved in the induction of phenylpropanoid and flavonoid biosynthesis [26,27]. UV-B radiation (290–320 nm) in sunlight results in damage to macromolecules in the plant cells, including DNA, due to the generation of reactive oxygen species [28]. Thus, phenolic and flavonoid compounds in plants, induced by UV-B exposure, function as UV-B-protecting compounds, which have a strong absorbance in the UV spectral range and powerful antioxidant activities [22].

In plants, sunlight leads to the acquisition of various secondary metabolites and serves as an important resource for photosynthesis [29]. Based on various previous studies, we investigated how the content of chemical components, such as phenolic and flavonoid compounds, varied in different samples in response to different environmental conditions. Accordingly, this result led to the hypothesis that the content of phenolics and flavonoids is affected by environmental conditions, such as the duration of sunlight irradiation and the harvesting time.

### 2.3. The Radical Scavenging Activity of S. plebeia Extracts is Influenced by the Growth Conditions

Two methods, 2,2′-diphenyl-1-picrylhydrazyl (DPPH) and 2,2′-azinobis(3-ethylbenzothiazoline-6-sulfonate (ABTS) radical scavenging activity, were employed to evaluate the antioxidant activity of the *S. plebeia* extracts obtained under different growing conditions. The DPPH and ABTS radical scavenging methods are commonly used to determine the antioxidant capacity of natural materials [30]. In Figure 3, the antiradical capacity of the sample extracts in the presence or absence of the UV wavelength on the light source were exhibited as the half-maximal inhibitory concentration (IC_50_) values. Of the *S. plebeia* extracts, the best antioxidant activity was shown in the six-month cultivated group (DPPH: 42.84 ± 0.10 µg/mL; ABTS: 53.83 ± 0.39 µg/mL), which was exposed in sunlight for two months. However, anti-inflammatory effects were not significantly different in the other treatment groups, and the anti-inflammatory activity of *S. plebeia* [4] might not be affected by above-mentioned conditions. Phenolics and flavonoids from natural plants exhibit promising radical scavenger properties [31,32]. Furthermore, among the compounds derived from *S. plebeia*, including phenolics, flavonoid glycosides, and their aglycones, it is reported that coniferyl aldehyde, hispidulin 7-glucuronide, hispidulin 7-glucoside, and 6-methoxyluteolin 7-glucoside possess powerful antioxidant activities [33]. Therefore, the highest antioxidant activity is in the extract obtained from the sunlight-irradiated cultivation (the six-month group), depending on the highly preserved content of the total phenolics and flavonoids, and this result showed a correlation between the sunlight exposure duration and the antioxidant activity as well as the phenolic and flavonoid levels, unless the sunlight exposure period was excessive.

### 2.4. HPLC Method Validation for Analyzing Oleanolic Acid and Ursolic Acid

An HPLC method for the quantitative determination of OA and UA in the *S. plebeia* sample was validated in terms of linearity, the limits of detection, quantification (LODs and LOQ), accuracy, and precision.

#### 2.4.1. Linearity

Linearity was examined with a standard solution mixed with OA and UA, and the linear range was from 0.005 to 0.05 mg/mL at five different concentration levels. Each calibration curve was performed in triplicate. The regression equations of OA and UA were *y* = 5417.6554*x* − 0.1599 and *y* = 4692.4373*x* + 0.1368, respectively (Table 2). Both of the correlation coefficients (*r*^2^) were >0.9999 within the tested range, and these showed a good linear regression.

#### 2.4.2. Limit of Detection (LOD) and Limit of Quantification (LOQ)

The LOD provides information on the detectable minimum level of the analyte, and the LOQ is the lowest amount of the analyte that can be quantified with an acceptable accuracy and precision. The LOD was determined as 0.5520 and 0.5458 μg/mL for OA and UA, and the LOQ was 1.6463 and 1.6783 μg/mL for OA and UA, respectively (Table 2).

#### 2.4.3. Recovery and Precision

The recovery test was carried out to investigate the effectiveness of this method. The *S. plebeia* samples were spiked with the mixture of OA and UA at three known concentrations (low, medium, high: 0.01, 0.05, 0.1 mg/mL, respectively), and the spiked samples were extracted and analyzed in accordance with the procedures described below. The average recoveries were expressed by calculating the ratio of the detected amount to the predicted amount (Table 3). The average percentage of the recoveries of the OA and UA were 95.1–104.8% and 97.2–107.1%, respectively. The intra- and inter-day variabilities were used to determine the precision of the developed method, and the variabilities are shown as relative standard deviations (RSDs) in the Table 3. Their RSD values were less than 2.0%, and this result implied that the established method corresponded to the recommendations of the International Conference on Harmonization (ICH) guidelines [34]. Therefore, the developed method is reliable and precise for the analysis of OA and UA in *S. plebeia*.

### 2.5. Quantitation of Oleanolic Acid and Ursolic Acid in S. plebeia under Different Cultivation Conditions

**Figure 4:**
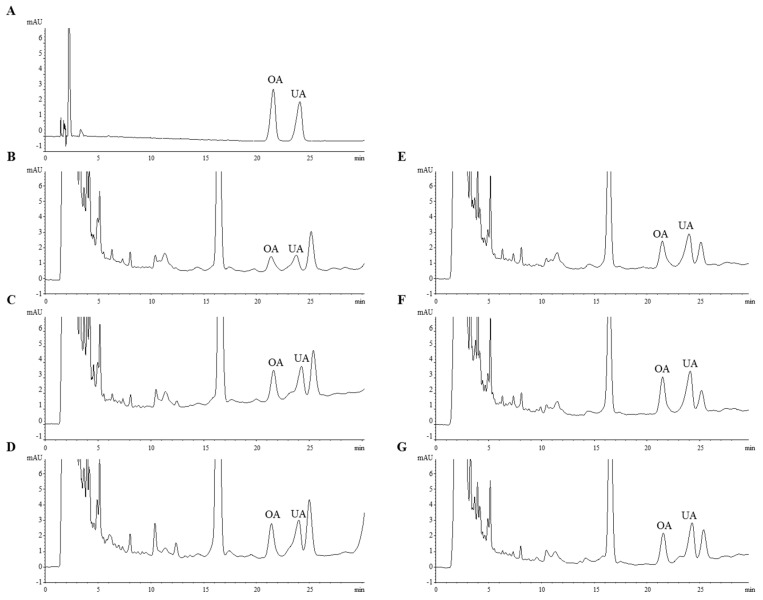
HPLC chromatograms (210 nm) of oleanolic acid (OA) and ursolic acid (UA) from *S. plebeia* (**A**): two triterpenoid standards; (**B**): 2 months; (**C**): 3 months; (**D**): 4 months; (**E**): 5 months; (**F**): 6 months; (**G**): 7 months.

The developed method was utilized for the quantitative analysis of OA and UA in the *S. plebeia* samples (Figure 4), which were influenced by sunlight and harvesting time. There was no significant difference in the OA and UA composition was observed compared to the content of phenolics and flavonoids; however, the results revealed that the two triterpenoids in the cultivated group (six months) with sunlight were higher than those in the other groups cultivated with fluorescent light (Table 4). Similarly, the triterpenoids composition on the sun-exposed side of the grapefruit was greater than that on the shade side [35], and this result was in good agreement with our experimental data.

Bioactive phytochemicals derived from the secondary metabolism of vegetables or medicinal plants contribute to various biological activities and protect a plant or an animal from the threat of infectious microorganisms, such as bacteria, viruses, and fungi [36]. In particular, OA and UA, which belong to the pentacyclic triterpenoid class, have been reported to have many bioactivities, such as anti-cancer, anti-inflammation, and antidiabetic activities [37,38]. OA and UA have similar physicochemical properties and coexist within a natural material, and numerous methods for analyzing these two constitutional isomers have been reported. Recently, a mixture of OA and UA was effectively separated by a simple reverse-phase HPLC system using a PAH polymeric C18 column [39]. For analyzing OA and UA in the *S. plebeia* extract, we slightly modified the HPLC analysis method established by Zhang et al., and the analysis of OA and UA in the *S. plebeia* samples using two different commercial PAH polymer C18 columns, the Eclipse PAH C18 column (5 μm, 4.6 × 250 mm, Agilent Technologies, Wilmington, DE, USA) and the Supelcosil™ LC-PAH HPLC column (5 μm, 4.6 × 250 mm Supelco, Bellefonte, PA, USA), showed similar resolutions. 

Although further studies are needed to identify the most important environmental factor for increasing bioactive triterpenoid content such as OA and UA, the source of sunlight may be one of the cultivation conditions involved in the processing of triterpenoid biosynthesis.

## 3. Materials and Methods

### 3.1. Plant Material

The seeds of *S. plebeia* and commercial horticultural substrates were purchased from a local store in Jeongeup, Korea. The seeds were washed with distilled water and germinated in the pots (550 × 270 × 50 mm). After one month, the sprouts were transplanted to the plant pot, and eighteen sprouts were divided into six experimental groups (*n* = 3) for harvesting at one-month intervals (2–7 months). The six groups were cultivated in a controlled room at 23 °C and with a mild humidity (70%) under the standard long-day condition (14 h/10 h light/dark in a day, a light intensity of 120 μmol·m^−2^·s^−1^) for a month. The first groups (2, 3, and 4 months) were maintained under the same culture conditions using fluorescent light sources, and the next groups (5, 6, and 7 months) were exposed to sunlight for a day length (sunrise to sunset) by maintaining the previous conditions except for the light source. All the experimental groups were washed with distilled water and dried in a thermostatic chamber at 23 °C and under low humidity (30%).

### 3.2. General Procedures

Spectroscopic data, such as UV, optical rotation, and NMR, were used to identify compounds isolated from *S. plebeia* and to measure antioxidant activity and composition of phenolics/flavonoids.

UV absorbance was measured with a Varioskan LUX (Thermo Fisher Scientific Inc., Waltham, MA, USA) spectrophotometer. Optical rotation was recorded on a Jasco P-2000 polarimeter (Jasco Corp., Tokyo, Japan). ^1^H- and ^13^C-NMR spectroscopic data were recorded on a JEOL JNM-EX400 (JEOL, Tokyo, Japan) instrument using TMS as a reference. HPLC-grade methanol, acetonitrile (J T Baker Chemicals, Center Valley, PA, USA), and glacial acetic acid (Merck, Darmstadt, Germany) were used in this study. The chemical compounds for the HPLC analysis were purchased from Sigma-Aldrich [caffeic acid, luteolin 7-glucoside, rosmarinic acid, luteolin, and oleanolic acid (St. Louis, Mo, USA)], Santa Cruz Biotechnology [hispidulin (Santa Cruz, CA, USA)], and TCI chemicals [ursolic acid (Tokyo, Japan)]. The dried and pulverized *S. plebeia* (30 kg) leaves were extracted with 95% ethanol at 70 °C for 5 h using an equipped extractor. The extract was concentrated *in vacuo* to yield 2.6 kg. The ethanol extract was suspended in deionized water and was partitioned with *n*-hexane, ethyl acetate, and H_2_O successively. The ethyl acetate fraction (541.2 g) was chromatographed on a silica gel column (Kieselgel 60, 230–400 mesh, Merck, Darmstadt, Germany) eluting with a step gradient of chloroform/methanol (1:0–0:1, *v*/*v*) to obtain 14 fractions. The 11th sub-fraction was separated by recrystallization in methanol, and the isolated crystal was further purified by preparative HPLC, which was rechromatographed on a Shimadzu LC-6AD (Shimadzu Co., Kyoto, Japan) instrument equipped with an SPD-20 A detector using a Phenomenex Luna C_18_ (Phenomenex Luna C_18_, 150 × 21.2 mm, 35% methanol) to give homoplantaginin (**5**, 208 mg). The NMR data were identified by a comparison to the reported data in the literature [10]. Yellow amorphous powder consisted of the following: ${\left[\alpha \right]}_{D}^{25}$ = −95.8 (*c* 0.1, CH_3_OH); ^1^H-NMR (400 MHz, DMSO-*d*_6_) δ_H_: 7.88 (2H, d, *J* = 8.8 Hz, H-2′,6′), 6.96 (1H, s, H-8), 6.92 (1H, d, *J* = 8.8 Hz, H-3′,5′), 6.65 (1H, s, H-3), 5.13 (1H, d, *J* = 7.2 Hz, H-1″), 3.96 (1H, dd, *J* = 12.0, 1.6 Hz, H-6a″), 3.89 (3H, s, 6-OCH_3_), 3.72 (1H, dd, *J* = 12.0, 6.0 Hz, H-6b″), 3.58 (1H, m, H-5″), 3.57 (1H, m, H-2″), 3.52 (1H, t, *J* = 9.2 Hz, H-3″), 3.42 (1H, t, *J* = 9.2, H-4); ^13^C-NMR (100 MHz, DMSO-*d*_6_) δ_C_: 184.5 (C-4), 167.1 (C-2), 163.4 (C-4′), 158.0 (C-7), 154.4 (C-9), 154.2 (C-5), 134.4 (C-6), 129.8 (C-2′,6′), 123.0 (C-1), 117.3 (C-3′,5′), 107.7 (C-10), 103.8 (C-3′), 102.2 (C-1″), 96.0 (C-8), 78.7 (C-3″),78.2 (C-5″), 74.9 (C-2″), 71.5 (C-4″), 62.7 (C-6″), 61.6 (6-OCH_3_).

### 3.3. Sample Preparation for HPLC Analysis

The aerial parts of the plants were pulverized (300 mg) and extracted in the methanol solvent (30 mL) using an ultrasonic apparatus for 2 h. After precipitating for 1 h, the supernatant was filtered through glass wool and the filtrate was evaporated *in vacuo*. The concentrate was completely dissolved in methanol (1 mL), and the residue was filtered using a syringe filter unit (PTFE 0.20 µm, Advantec, Tokyo, Japan) prior to the HPLC analysis.

### 3.4. Flavonoid and Phenolic Compound Quantification

The compounds were analyzed with a Shimadzu HPLC system on a Phenomenex C_18_ Gemini column (5 μm, 4.6 × 250 mm) equipped with a binary pump (SPD-20AD), a UV detector (SPD-20A), an autosampler (SIL-20A), a column oven (CTO-20AC), a degasser, (DGU-20A_3_) and an LC solution system (Shimadzu, Kyoto, Japan). The flavonoid and phenolic compound quantification was conducted using a calibration curve at ten concentrations over a linear range (0.0005–1.0 mg/mL), and the chromatographic analysis was performed using the method previously reported by Jin et al. [13].

### 3.5. Determination of the Total Phenolic and Flavonoid Contents

A method described by Dewanto et al. [40] was slightly modified for determining the total phenolic and flavonoid contents. Briefly, the sample was dissolved at a 1 mg/mL concentration in methanol, and a 12.5 μL aliquot of the sample solution was added to 12.5 μL of the Folin-Ciocalteu reagent and 50.0 μL of deionized water. After 6 min, 125 μL of 7% sodium carbonate (Na_2_CO_3_) and 100 μL of deionized water were mixed with the solution and incubated at ambient temperature for 90 min. The absorbance values of the sample solution were measured at 760 nm using a microplate reader (Thermo Fisher Scientific Inc., Waltham, MA, USA). The total phenolic content is expressed in mg of gallic acid equivalents (GAE/g of dried mass, DM) and is based on the standard curve of gallic acid. The calibration curve range was 0.0–0.2 mg/mL at six concentrations (*R* = 0.999). The flavonoid quantification is expressed as mg/quercetin equivalents (QE/g of dried mass, DM), and it was determined using a standard curve with quercetin (Sigma-Aldrich, St. Louis, MO, USA) ranging from 0.0 to 0.2 mg/mL at six concentrations (*R* = 0.999). The sample was dissolved at a 1 mg/mL concentration in methanol, and a 25 μL aliquot of the sample solution and 7.5 μL of 5% sodium nitrite (NaNO_2_) were mixed in the 96-well microplate. After 6 min, 50 μL of 1 M sodium hydroxide (NaOH), 15 μL of 10% aluminum chloride (AlCl_3_), and 152.5 μL of distilled water were added to the mixture. The absorbance values of each sample solution were measured at 510 nm.

### 3.6. Antioxidant Activity

The antioxidant capacity of the samples was analyzed using previously reported DPPH and ABTS radical scavenging methods [32,41]. Briefly, a 0.2 mM DPPH ethanolic solution (100 μL) and the samples, diluted in a range from 0.0 to 0.2 mg/mL in methanol (100 μL), were mixed at a 1:1 ratio (*v*/*v*). The absorbance at 517 nm was recorded on a microplate reader (Thermo Fisher Scientific). In addition, 7 mM ABTS and 2.4 mM potassium persulfate (K_2_S_2_O_8_) were dissolved in distilled water, respectively. The mixture of 7 mM ABTS and 2.4 mM K_2_S_2_O_8_ at a ratio of 1:1 (*v*/*v*) was incubated in the dark at ambient temperature for 24 h to generate ABTS radical cations (ABTS^+^). The ABTS stock solution was diluted with distilled water to adjust the absorbance to approximately 0.700 at 734 nm. Next, 100 μL of the ABTS stock solution and 100 μL of the sample solution (0.0–0.2 mg/mL) in methanol were incubated for 2 min, and the absorbance at 734 nm was measured using a microplate reader (Thermo Fisher Scientific). The radical scavenging activities were calculated as a percentage using the following equation:

Scavenging activity = (1 − *A_S_*/*A_0_*) × 100

where *A_0_* and *A_S_* are the signal intensities of the blank and sample, respectively. The radical scavenging activity of the sample was expressed as the half-maximal inhibitory concentration (IC_50_) values.

### 3.7. Cell Culture

The macrophage cell line RAW 264.7 (TIB-71, ATCC, Manassas, VA, USA) was cultivated in DMEM (Gibco, Grand Island, NY, USA) supplemented with 2 mM glutamine, 100 units/mL penicillin, 100 μg/mL streptomycin (1% Antibiotic-Antimycotic^TM^, Invitrogen, Carlsbad, CA, USA), and 10% non-heat-inactivated fetal bovine serum (Gibco, 16000-044, Grand Island, NY, USA) under standard cell culture conditions (5% CO_2_ at 37 °C).

### 3.8. Nitric Oxide Production Measurements in Macrophage Cells

The amount of stable nitrite, which is the final nitric oxide product generated in LPS-stimulated macrophages, was determined with a colorimetric assay after 24 h of cell seeding (1 × 10^5^ cells/well in 96-well plate). Briefly, 100 μL of the culture supernatant was mixed with an equal volume of Griess reagent (1% sulfanilamide, 0.1% naphthylethylenediamine dihydrochloride, 2.5% H_3_PO_4_) and incubated at room temperature for 10 min. The absorbance at 550 nm was read using a spectrophotometer.

### 3.9. Method Validation for Analyzing Oleanolic Acid and Ursolic Acid

A previously established method by Zhang et al. [39] was modified for analyzing two triterpenes: oleanolic acid and ursolic acid. Briefly, the Agilent 1200 HPLC system (Agilent Technologies, Wilmington, DE, USA) was equipped with a quaternary pump (G1311A), a degasser (G1322A), an autosampler (G1329A), a column oven (G1316A), and a diode array detector (G1315D) at 190–400 nm wavelength. A polycyclic aromatic hydrocarbons (PAHs, 5 μm, 4.6 × 250 mm, Eclipse PAH, Agilent Technologies, Wilmington, DE, USA) column, maintained at 30 °C, was used, eluted with a gradient of water (A, H_2_O) and acetonitrile (B, CH_3_CN). The solvent gradient was from 71 to 76% of CH_3_CN within 30 min at a flow rate of 1 mL/min.

On the basis of the International Conference on Harmonization (ICH) guidelines [34], the HPLC analysis method was validated with respect to the limit of detection (LOD), the limit of quantification (LOQ), linearity, accuracy, and precision. The mixture of oleanolic acid and ursolic acid (0.1 mg/mL), at the rate of 1:1(*v*/*v*), was diluted to generate a calibration curve. The linear regression equation was calculated using three calibration curves at five different concentrations ranging from 5 to 50 ppm. The LOD and LOQ were determined by calculating the lowest detectable peak in the chromatogram, which had signal-to-noise (S/N) ratios of 3 and 10, respectively.

The accuracy of the method was evaluated via the recovery test in triplicate at three different concentrations (0.01–0.1 mg/mL), corresponding to oleanolic acid and ursolic acid, which were spiked in each sample. The recovery rates (%) were determined by calculating the ratio of the detected amount to the spiked sample and the expected amount.

The precision test of the method was assessed according to the intra-day and inter-day variability for the compounds. The intra-day precision test was analyzed in triplicate at three concentration levels (0.01–0.1 mg/mL) during a day. The inter-day precision test was performed in triplicate for each sample at three concentration levels (0.01–0.1 mg/mL) during sequential days. The results are expressed as the relative standard deviation (RSD, %) The method was considered precise if the RSD was within ±2%.

### 3.10. Statistical Analysis

Excel^®^ software was used for the statistical analyses, and the data are expressed as the means ± standard error (S.E.).

## 4. Conclusions

For developing drug derived from natural sources, the pharmaceutical industry requires that a raw material be consistently maintained with an identical quality to the extent possible and exhibit suitable biological activity without side effects.

Environmental conditions, such as sunlight, may affect the chemical composition, antioxidant activity, and appearance of *S. plebeia*. Among the metabolites of the plant, the phenolic, flavonoid, and triterpenoid compounds showed many differences in the sun-exposed *S. plebeia* groups. Therefore, we concluded that the phytochemical constitution of *S. plebeia* was correlated with the environmental conditions, including the duration of sunlight irradiation, and the secondary metabolites. Nevertheless, additional research is needed to investigate whether the variance in the chemical composition was affected by the sunlight or growing period, and our studies may provide useful information for controlling the quality of *S. plebeia*.

## Figures and Tables

**Table 1:** Content of the phenolic and flavonoid compounds in the *S. plebeia* extracts in the different cultivation areas.

Samples	Cultivation Area	Analytes (mg/g Extract)						Total
		Caffeic Acid	Luteolin 7-Glucoside	Rosmarinic Acid	Homoplantaginin	Luteolin	Hispidulin	
KSPA1	Jeju	1.19 ± 0.01	7.24 ± 0.15	63.98 ± 0.45	29.27 ± 0.20	1.46 ± 0.02	2.75 ± 0.02	105.88
KSPB1	Gyeonggi	1.02 ± 0.02	3.90 ± 0.02	120.65 ± 2.48	27.50 ± 0.68	0.66 ± 0.01	0.91 ± 0.01	154.63
KSPB2	Gyeonggi	0.95 ± 0.01	9.19 ± 0.16	94.06 ± 2.25	28.99 ± 0.55	0.91 ± 0.05	1.62 ± 0.02	135.72
KSPC1	Kangwon	0.58 ± 0.02	21.82 ± 0.02	60.36 ± 0.09	34.70 ± 0.24	0.70 ± 0.01	1.06 ± 0.01	119.22
KSPD1	Chungcheong	0.58 ± 0.02	10.30 ± 0.25	45.70 ± 4.19	28.47 ± 0.29	0.63 ± 0.01	1.02 ± 0.01	86.70
KSPD2	Chungcheong	0.60 ± 0.01	11.81 ± 0.17	27.81 ± 1.65	26.40 ± 0.73	0.65 ± 0.01	0.80 ± 0.01	68.08
KSPD3	Chungcheong	0.96 ± 0.04	4.07 ± 0.30	119.80 ± 8.11	16.36 ± 1.34	0.63 ± 0.01	0.90 ± 0.03	142.73
KSPD4	Chungcheong	0.89 ± 0.04	6.48 ± 0.06	138.99 ± 1.86	17.83 ± 0.26	0.62 ± 0.01	0.89 ± 0.03	165.70
KSPE1	Jeolla	0.60 ± 0.03	7.79 ± 1.43	113.81 ± 20.18	27.71 ± 4.97	0.63 ± 0.01	0.90 ± 0.03	151.43
KSPE2	Jeolla	0.63 ± 0.02	9.95 ± 0.17	106.68 ± 1.06	33.50 ± 0.34	0.71 ± 0.03	1.16 ± 0.01	152.63
KSPE3	Jeolla	0.83 ± 0.02	2.80 ± 0.13	44.35 ± 0.86	13.92 ± 0.57	0.77 ± 0.01	1.48 ± 0.03.	64.15
KSPF1	Gyeongsang	1.16 ± 0.07	4.41 ± 0.45	110.61 ± 14.33	16.39 ± 1.86	0.68 ± 0.01	1.12 ± 0.06	134.36
KSPF2	Gyeongsang	0.88 ± 0.04	3.88 ± 0.08	130.89 ± 1.61	15.81 ± 0.65	0.63 ± 0.01	1.80 ± 0.01	152.89
KSPF3	Gyeongsang	0.88 ± 0.02	1.89 ± 0.04	65.09 ± 0.76	6.57 ± 0.25	0.62 ± 0.01	0.92 ± 0.02	75.97
KSPF4	Gyeongsang	0.64 ± 0.01	21.45 ± 1.20	80.48 ± 3.38	46.23 ± 1.74	0.80 ± 0.02	1.31 ± 0.05	150.91
KSPF5	Gyeongsang	0.90 ± 0.01	2.81 ± 0.09	82.34 ± 4.19	10.09 ± 0.42	0.62 ± 0.01	0.91 ± 0.05	97.68
KSPF6	Gyeongsang	0.56 ± 0.02	4.98 ± 0.36	66.56 ± 4.51	36.08 ± 2.39	1.57 ± 0.10	2.79 ± 0.15	112.54
KSPF7	Gyeongsang	0.65 ± 0.02	33.28 ± 2.17	92.21 ± 6.28	53.13 ± 4.66	0.82 ± 0.02	1.25 ± 0.03	181.33

Each *S. plebeia* sample was extracted in a methanol solvent using ultra-sonication extraction for 2 h, and the extracts were analyzed by the established methods. The values represent the mean ± S.E. from triplicate experiments.

**Table 2:** Linearity, LOD, and LOQ of oleanolic acid and ursolic acid.

Compound	*t_R_* (min)	Equation (Linear Model) *^a^*	Linear Range (mg/mL)	*r*^2^ *^b^*	LOD *^c^* (μg/mL)	LOQ *^d^* (μg/mL)
Oleanolic acid	21.35	*y* = 5417.6554*x* − 0.1599	0.005–0.05	0.9999	0.5520	1.6463
Ursolic acid	24.04	*y* = 4692.4373*x* + 0.1368	0.005–0.05	0.9999	0.5458	1.6783

*^a^* y: peak area at 210 nm; x: standard concentration (mg/mL); *^b^ r*^2^: coefficient of determination with 5 indicated points in the calibration curves; *^c^* LOD: limit of detection, S/N = 3 (*n* = 6); *^d^* LOQ: limit of quantification, S/N = 10 (*n* = 6).

**Table 3:** Accuracy and the intra- and inter-day precision of oleanolic acid and ursolic acid.

Compound	Sample	Spiked Amount (mg/mL)	Recovery Test (%, *n* = 3)	Precision Test (*n* = 3)	
				Intra-Day RSD *^a^* (%)	Inter-Day RSD (%)
Oleanolic acid	2 Months	0.01	104.8	0.06	0.60
		0.05	100.4	0.11	0.29
		0.1	100.5	0.06	0.51
	3 Months	0.01	99.0	0.00	0.27
		0.05	99.5	0.21	0.87
		0.1	97.9	0.13	0.21
	4 Months	0.01	98.3	0.24	0.62
		0.05	99.5	0.21	0.37
		0.1	99.7	0.12	0.47
	5 Months	0.01	103.0	0.03	0.24
		0.05	100.6	0.12	1.15
		0.1	99.5	0.10	0.96
	6 Months	0.01	101.8	0.09	0.41
		0.05	100.3	0.68	1.00
		0.1	100.9	0.05	0.68
	7 Months	0.01	97.7	0.09	0.88
		0.05	95.1	0.23	1.04
		0.1	98.7	0.07	0.72
Ursolic acid	2 Months	0.01	101.6	0.30	0.81
		0.05	100.6	0.01	0.98
		0.1	99.4	0.09	1.51
	3 Months	0.01	100.3	0.03	0.14
		0.05	101.6	0.50	0.13
		0.1	99.3	0.25	0.85
	4 Month	0.01	105.5	0.30	0.28
		0.05	98.1	0.10	0.86
		0.1	100.3	0.23	0.38
	5 Months	0.01	102.8	0.07	0.38
		0.05	107.1	0.04	0.35
		0.1	98.9	0.11	0.68
	6 Months	0.01	100.8	0.11	0.35
		0.05	99.6	0.30	0.10
		0.1	98.3	0.02	0.57
	7 Months	0.01	97.9	0.05	0.53
		0.05	97.2	0.25	1.72
		0.1	99.1	0.03	0.63

*^a^* RSD: relative standard deviation.

**Table 4:** Oleanolic acid and ursolic acid content in *S. plebeia* at the different growing periods.

Samples	2 Months	3 Months	4 Months	5 Months	6 Months	7 Months
	Contents (*n* = 3)					
Oleanolic Acid (mg/g)	0.766 ± 0.003 *^a^*	1.246 ± 0.002	1.433 ± 0.015	1.270 ± 0.019	1.546 ± 0.007	1.546 ± 0.027
Ursolic Acid (mg/g)	0.915 ± 0.008	1.270 ± 0.006	1.376 ± 0.016	1.604 ± 0.022	2.066 ± 0.037	1.677 ± 0.003

*^a^* Standard error (mg/g).
